# Selective Phosphorylation of Akt/Protein-Kinase B Isoforms in Response to Dietary Cues

**DOI:** 10.3389/fcell.2019.00206

**Published:** 2019-10-10

**Authors:** Laura Christin Trautenberg, Elodie Prince, Cornelia Maas, Nora Beier, Freya Honold, Michal Grzybek, Marko Brankatschk

**Affiliations:** ^1^TU Dresden (BIOTEC), Dresden, Germany; ^2^Paul Langerhans Institute Dresden of the Helmholtz Zentrum München at the University Hospital and Faculty of Medicine Carl Gustav Carus of TU Dresden, Technische Universität Dresden, Dresden, Germany; ^3^German Center for Diabetes Research (DZD e.V.), Oberschleissheim, Germany

**Keywords:** *Drosophila*, *Saccharomyces cerevisiae*, *Cystobasidium oligophagum*, Akt, PKB, Akt phosphorylation, insulin signaling, yeast lipids

## Abstract

A calorie-rich diet is one reason for the continuous spread of metabolic syndromes in western societies. Smart food design is one powerful tool to prevent metabolic stress, and the search for suitable bioactive additives is a continuous task. The nutrient-sensing insulin pathway is an evolutionary conserved mechanism that plays an important role in metabolism, growth and development. Recently, lipid cues capable to stimulate insulin signaling were identified. However, the mechanistic base of their activity remains obscure to date. We show that specific Akt/Protein-kinase B isoforms are responsive to different calorie-rich diets, and potentiate the activity of the cellular insulin cascade. Our data add a new dimension to existing models and position *Drosophila* as a powerful tool to study the relation between dietary lipid cues and the insulin-induced cellular signal pathway.

## Introduction

Food composition is instructive for the metabolic response of organisms and recent studies demonstrate an important role of lipid cues including dietary lipids in modulating systemic insulin signaling. The identity and mechanism of such metabolically active lipids remains obscure; however, their regulating function is restricted to calorie-rich nutritional settings and is second to the role of sugars (Migrenne et al., 2006; Oh et al., 2010; Brankatschk et al., 2014). It is suggested that circulating fatty acids bind to cellular receptors and that way, induce the secretion of metabolic regulators (Nolan et al., 2006; Oh et al., 2010; Hauke et al., 2018). On the other hand, absorbed lipids are re-integrated into cellular membranes and therefore, can change biophysical membrane properties (Abbott et al., 2012). Variables like membrane fluidity and thickness possibly modulate the amount and activity of membrane proteins such as the Insulin receptor (Ginsberg et al., 1981; Murphy, 1990; Gutmann et al., 2018). The Insulin receptor (InR) is a dimeric type-I membrane protein that belongs to the tyrosine-kinase receptor family (Fernandez et al., 1995). Bound to insulin, the InR recruits adapter proteins and activates the PI-3 kinase (Böhni et al., 1999). The PI-3 kinase converts the inner leaflet membrane lipid PI(4,5)P_2_ into PI(3,4,5)P_3_, which attracts the Protein kinase B/Akt (Scanga et al., 2000). Different Akt isoforms have been identified in vertebrates and invertebrates. For instance, mice or fruit flies express three different protein versions (Andjelković et al., 1995; Gonzalez and McGraw, 2009a). In *Drosophila*, the presence of individual Akt isoforms (dAkt's) is stage-dependent. Adult flies express two dAkt proteins, which are different in size: the smaller dAkt^66^ and the larger dAkt^85^, close to 66 and 85 kDa, respectively (Andjelković et al., 1995). The structure of Akt shows high molecular and functional conservation; this kinase possesses multiple regulatory phosphorylation sites and the positions dAkt^Ser505^ and dAkt^Thr342^ are characterized (Manning and Toker, 2017). The functional relevance of the vertebrate Akt1^Ser473^ (which corresponds to the dAkt^Ser505^) is well-studied (Alessi et al., 1996); however, the biological role of Akt1^Thr308^ (which corresponds to the dAkt^Thr342^) is more obscure. It is widely accepted that the enzyme reaches full activity once phosphorylated on both positions (Alessi et al., 1996; Scheid et al., 2002). Akt is a negative regulator of the transcription factor FOXO (Calnan and Brunet, 2008). When insulin signaling levels are low, FOXO translocates from the cell cytoplasm into the nucleus (Puig, 2003). Whereas, high Akt activity prevents FOXO from entering the nucleus, and the cells predominantly switch to anabolic reactions building stocks of storage molecules such as fatty acids (Xu et al., 2012). Taken together, circulating lipids are potentially capable to modulate the insulin-signaling cascade at multiple levels. If so, what pressures could possibly favor lipids as regulators of the insulin signaling?

*Drosophila* feed preferentially on rotting fruits, a diet composed by plant material and microbes such as yeast. Fruits are the main source for carbohydrates while microbes provide dietary amino acids. It was shown that a calorie-rich diet supplemented with yeast lipids increases circulating *Drosophila* insulin-like peptides (dILPs) and facilitates high systemic insulin signaling levels (Brankatschk et al., 2014). High insulin signaling stimulates the proliferation and developmental rate of flies. On the other hand, the fly dietary lipid composition is dependent on the consumed yeast species, microbial growth stage and available carbon source, and environmental temperature (Chandler et al., 2012; Klose et al., 2012). Decomposing plant material is rich in sugars and proteins, which are degraded to smaller molecules (monosaccharides and amino acids) prior to intestinal absorption. Although it is shown that extreme quantities or the absence of individual compounds in experimental conditions can change the activity of metabolic circuits, such nutritional settings are not likely found in the wild.

The structure of fatty acids and other lipid species is defined by their origin. For instance, plants produce more unsaturated and long fatty acids, as well as phytosterols which are structurally different from fungal or mammalian counterparts. If microbial lipid cues convey dietary signals to flies, then two principal questions arise. First, do all microbes associated with *Drosophila* promote the proliferation of fruit flies? Second, is the growth dependent microbial lipid composition instructive for the generative cycle of flies?

Here, we report the isolation of *Cystobasidium oligophagum*, a ubiquitous *Basidiomycota*, from *Drosophila* droppings. Although *C. oligophagum* attracts adult flies, we show that these yeasts do not promote fruit fly oviposition or development. Compared to baker's yeast (*Saccharomyces cerevisiae*), *C. oligophagum* produce similar amounts of protein and sugar but differ in their lipid composition. Calorie-rich food based on either yeast type supports the generative cycle of *Drosophila*; however, only diet manufactured from stationary *S. cerevisiae* accelerates its developmental rate and increases egg production. Moreover, we demonstrate that flies kept on food prepared from stationary *S. cerevisiae* upregulate selectively the dAkt^85^-isoform, and that the enzyme is highly phosphorylated. Thus, we speculate that lipid cues derived from stationary *S. cerevisiae* stimulate the accumulation of PI(3,4,5)P_3_ at the inner plasma-membrane leaflet of *Drosophila* cells. dAkt^85^ binds to PI(3,4,5)P_3_ and as such is accessible for the phosphoinositide-dependent Protein kinase 1 (PDK1) or the Rictor-mammalian target of rapamycin complex 2 (mTorC2) (Cho et al., 2001; Sarbassov et al., 2005).

## Results

### *Cystobasidium oligophagum* and *Saccharomyces cerevisiae* Have Different Lipid Qualities

**Figure 1:**
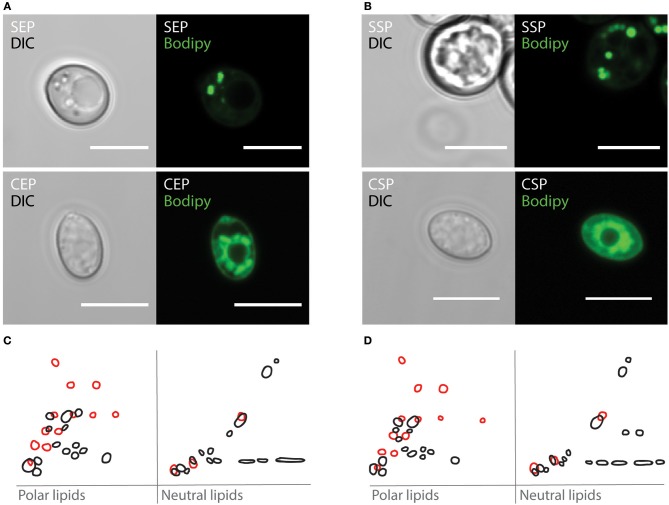
*Cystobasidium oligophagum* and *Saccharomyces cerevisiae* have different lipid profiles. **(A,B)** Photographs of yeast harvested at specific growth stages probed with BODIPY-505. Shown are *S. cerevisiae* (S) or *C. oligophagum* (C) in exponential (EP) in **(A)** or stationary growth phase (SP) in **(B)**. Scale bar = 5μm. **(C,D)** Shown are 2D-TLC profiles of polar and neutral lipids. Lipid signatures from *S. cerevisiae* (black) or *C. oligophagum* (red) from exponential **(C)** and stationary **(D)** growth stages.

On rotting plant material, yeast are predominant microbes preferred by *Drosophila*. To investigate which fungi are associated with flies, we have analyzed microbial isolates from fly droppings of wild type *OregonR* flies kept on plant food in an open cage. Fly poo positioned on plant food at 20°C contained *C. oligophagum*; but not *S. cerevisiae*, which is the preferred yeast used in experimental *Drosophila* research. To compare the caloric value of each yeasts, we have cultivated *C. oligophagum* or *S. cerevisiae* at 20°C in a lipid-free, defined medium. We harvested the fungi in their exponential (EP) and stationary growth phases (SP), and measured their protein and trehalose content using commercial detection assays (Supplementary Figure 1). Remarkably, both fungi produce very similar amounts of protein and trehalose (Supplementary Figures 1B,D). To evaluate the fungal lipidomes, we extracted lipids from all four yeast samples and adjusted their total lipid amounts. Subsequently, we separated individual lipid classes by using reverse phase Thin-Layer Chromatography (2D-TLC). As expected, we confirmed that *S. cerevisiae* lipid profiles depend on growth stages (Figures 1C,D in black and Supplementary Figure 1E; Klose et al., 2012). Interestingly, lipids from *C. oligophagum* show minimal growth-dependent quality changes (Figures 1C,D in red and Supplementary Figure 1E). To estimate relative lipid quantities, we have adjusted the lipid amounts to phosphate levels measured in our samples. Like reported, the lipidome of *C. oligophagum* is overrepresented by lipids with properties shown by free fatty acids with respect to *S. cerevisiae* (Vyas and Chhabra, 2017). Furthermore, we found that the relative amounts of individual lipid classes do not vary between EP and SP stages. In contrast, proliferating *S. cerevisiae* have less triacylglycerids (TAGs) and sterol-esters with respect to stationary cells (data not shown). To visualize the lipid distribution in fungal cells, we stained live yeasts with Bodipy-505. The dye is capable to penetrate the plasma membrane and preferentially accumulates in lipid-rich regions such as lipid droplets. As expected, in *S. cerevisiae* cells, Bodipy-505 is enriched in lipid droplets and we noted that SP yeast cells contain a higher number of such organelles (Figures 1A,B). Taken together, we have confirmed that lipid extract qualities and membrane organizations of *S. cerevisiae* depend on growth; whereas *C. oligophagum* lipid profiles appear remarkably static throughout the generative cycle.

### Designed Yeast Food Mimics the Biological Activity of the Respective Yeast

**Figure 2:**
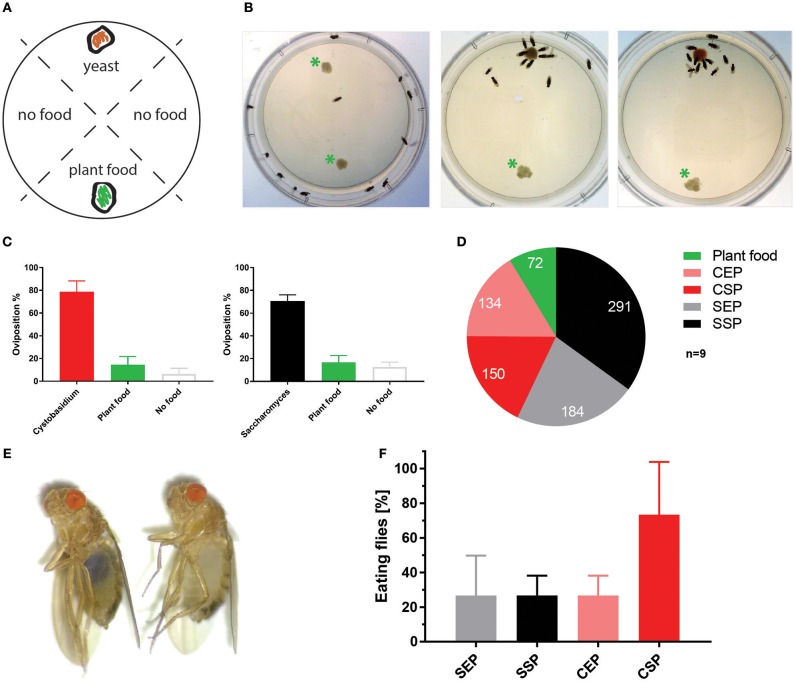
*Cystobasidium oligophagum* attracts *Drosophila melanogaster*. **(A)** Scheme that depicts our quantification approach. Assay plates are divided into four sectors loaded with food (yeast or plant food) or without a bait (no food). **(B)** Shown are screenshots from movies that show the feeding behavior of wild type flies. Photograph with control plate (left), plates testing *S. cerevisiae* (middle), or *C. oligophagum* (right). *Plant food bait. **(C)** Plotted are percentages of eggs positioned in different food sectors: *S. cerevisiae* (black), *C. oligophagum* (red), plant food (yellow), or no food (gray). **(D)** Depicted are total egg numbers (*n* = 9/food type) from flies fed with plant food, exponentially (EP), and stationary (SP) grown *S. cerevisiae* (S) or *C. oligophagum* (C). **(E)** Shown is a photograph of a fly feeding on blue-stained food (left) and a not feeding fly kept on the identical diet (right). **(F)** Plotted is the percentage of feeding wild type flies (*n* = 3, total of 15 mated females/food type) kept on different blue stained diets. Note, there is no significant difference between CSP and other samples (Dunn's multiple comparisons test: *p* > 0.05).

To test if *C. oligophagum* attract adult flies, we placed wild-type *OregonR* in feeding chambers and video-recorded their feeding behavior. Already after a short adaptation time, all tested animals started to feed and females positioned their eggs close to the provided yeast bait (Figures 2A–C, Movies 1–3). Interestingly, we noted that flies feeding on *S. cerevisiae* produced reproducibly higher egg numbers compared to flies feeding on *C. oligophagum* or plant material only (*n* = 5, total egg number: *C. oligophagum* = 299, *S. cerevisiae* = 578, and plant material = 184 eggs, each assay plate with 20–30 females and 15 males at 20°C). The oviposition rate of females is one readout for their metabolic activity. Since both yeast types do not show gross differences in their sugar or protein amounts, we speculated either that flies feed less on *C. oligophagum* or that dietary lipid profiles are instructive for the fly metabolism.

To neglect possible caloric differences or problems with the intestinal accessibility of nutrients we designed fly food recipes based on our cultured yeast. All four diets are equi-caloric (~550 kcal/l), enriched in plant proteins and sugars to prevent carbon or amino acid shortages, and feature approximately identical carbohydrate: protein ratios (2:1). Moreover, the nature of our recipes minimizes the proportion of native yeast sugars and proteins (native yeast sugar and proteins are ~0.0002% w/v while added sugar and proteins are ~0.11% w/v in food). On the other hand, the fungal lipidomes are the only lipid source. To estimate the quantity of lipids, we weighted the dry mass of each food type and measured phosphate amounts present in respective lipid extracts. Food (F) produced from *C. oligophagum* (C) or *S. cerevisiae* (S) show similar phospholipid mass (*n* = 3, CEP-F = 0.6; CSP-F = 0.5, SEP-F = 0.3, and SSP-F = 0.4 mg phospholipids/g food). Of note, the assay is unable to capture neutral lipids such as fatty acids and sterols. In addition, the unavoidable heat treatment during food preparation could break or modify some yeast products. Therefore, we have analyzed the phosphate-standardized lipid extracts using TLC. We found that heat treatment is changing the TLC profile of yeast. Especially some hydrophobic compounds produced by *C. oligophagum* with properties similar to neutral lipids (e.g., fatty acids, diacylglycerids, TAGs) are degraded at 110°C; whereas lipids from *S. cerevisiae* proof to be more heat stable (Supplementary Figure 2). At this point, we are not able to identify the heat-sensitive molecules. Taken together, the lipid profiles of the designed diets differ from respective yeast lipidomes.

**Figure 3:**
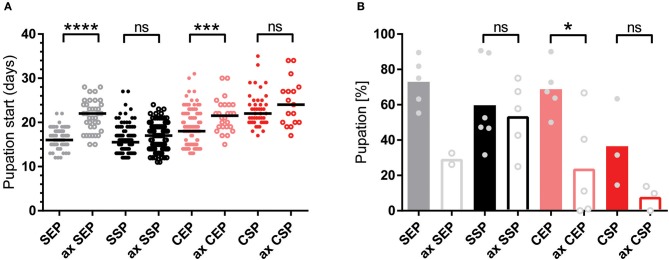
Stationary yeast food rescues the metabolic syndrome of axenic larvae. **(A,B)** Plotted is the larval developmental speed **(A)** and survival rate **(B)** of axenic (ax) and microbe-associated larvae kept on food based on exponential (EP) and stationary (SP) *S. cerevisiae* (S) or *C. oligophagum* (C). Each spot represents one tracked individual **(A)** or one experimental cohort of minimum 20 individuals **(B)**. Differences between the mean values of experimental groups were compared with Dunn's multiple comparisons test for developmental speed and Tukey's multiple comparison test for survival rate data. n.s., none significant; *p < 0.05, ***p < 0.001, ****p < 0.0001. *P*-values: **(A)** CEP vs. ax CEP *p* = 0.0009, CEP vs. CSP *p* < 0.0001, CEP vs. ax CSP *p* = 0.0001, CEP vs. SEP *p* = 0.0091, CEP vs. ax SEP *p* = 0.0001, CEP vs. SSP *p* < 0.0001, ax CEP vs. SEP *p* < 0.0001, ax CEP vs. SSP *p* < 0.0001, ax CEP vs. ax SSP *p* < 0.0001, CSP vs. SEP *p* < 0.0001, CSP vs. SSP *p* < 0.0001, CSP vs. ax SSP *p* < 0.0001, ax CSP vs. SEP *p* < 0.0001, ax CSP vs. SSP *p* < 0.0001, ax CSP vs. ax SSP *p* < 0.0001, SEP vs. ax SEP *p* < 0.0001, ax SEP vs. SSP *p* < 0.0001, ax SEP vs. ax SSP *p* < 0.0001; (B) CEP vs. ax CEP *p* = 0.0336, CEP vs. ax CSP *p* = 0.008, ax CEP vs. SEP *p* = 0.0158, ax CSP vs. SEP *p* = 0.004, ax CSP vs. SSP *p* = 0.0261.

To test if the created yeast food recipes induce similar physiological changes in feeding *Drosophila*, we have repeated the feeding-behavior experiments. Given a choice between plant material and yeast-based diets, adult flies tended to feed on the latter. Moreover, feeding was indifferent between two tested wild type genotypes irrespective of the provided yeast-food type (Figures 2E,F). Female flies kept on SSP-F produced more eggs than siblings kept on the other food types, thus mirroring the activity of live *S. cerevisiae* (Figure 2D, Movies 4–7). To investigate if food or fly-associated microbes convey the biological activity of SSP-F, we created microbe-free (axenic) larvae and tracked their growth and survival rates, both indicators for metabolic activity. Axenic animals rely entirely on provided food compositions and show a direct response to nutritional settings. Interestingly, only larvae kept on SSP-F were able to match the developmental speed and success of their microbe-bearing counterparts. All other axenic cultures developed slower and with lower survival rates (Figure 3).

Taken together, the designed food types are indifferent in their caloric content, their carbohydrate: protein ratios are identical, and the diets are rich in amino acids and monosaccharides. In consequence, the quality and quantity of the respective dietary lipid load is modulating the response of flies.

### Dietary Lipid Extracts Regulate the Cellular Insulin Signal Cascade

**Figure 4:**
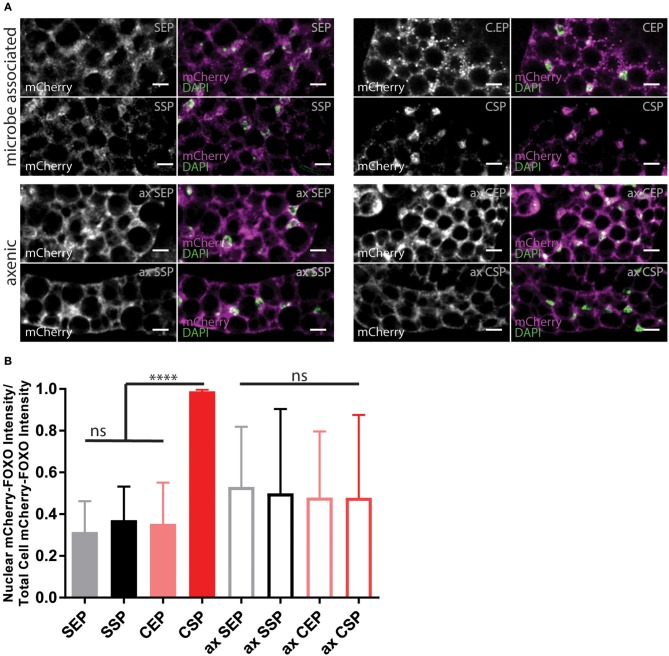
Yeast food quality and microbial load modulate dFOXO activity. **(A)** Photographs from larval fat bodies (early 3rd instar larvae). Samples were probed for mCherry-FOXO (magenta, white) and DAPI (green). Shown are samples from microbe associated (upper two panels) or axenic (ax, lower two panels) individuals kept on food based on stationary (SP) or exponentially (EP) grown *S. cerevisiae* (S) or *C. oligophagum* (C). Scale bars = 10μm. **(B)** Plotted is the quantification of mCherry-FOXO fluorescent intensity in nuclei of fat body cells (*n* ≥ 3 with ≥15 cells/sample). Differences between the mean values of groups were compared with Tukey's multiple comparisons test. n.s., none significant; ****p < 0.0001. *P*-values: SEP vs. CSP *p* < 0.0001, SEP vs. ax SEP *p* = 0.0019, SEP vs. ax SSP *p* = 0.0275, SSP vs. CSP *p* < 0.0001, CEP vs. CSP *p* < 0.0001, CEP vs. ax SEP *p* = 0.0192, CSP vs. ax SEP *p* < 0.0001, CSP vs. ax SSP *p* < 0.0001, CSP vs. ax CEP *p* < 0.0001, CSP vs. ax CSP *p* < 0.0001.

Animals fed with high-fat diet or kept on chow-food show very different lipidomes (Carvalho et al., 2012). We have shown that the lipid extract composition of the different yeast foods varies. Thus, we wondered if the nutritional lipid quality in the experimental setups would induce endogenous lipid changes in feeding *Drosophila* (Carvalho et al., 2012). To do so, we transferred adults reared on normal food and kept the animals for 7 or 14 days on our yeast diets. Lipid extracts from larvae or adult fly heads did not reveal changes in the composition of complex endogenous lipids at 1D-TLC resolution (Supplementary Figure 3). It remains possible that structural qualities of absorbed and integrated dietary fatty acids, only visible by mass spectroscopy, regulate the fecundity and developmental rate. In addition, it was shown that *S. cerevisiae* are able to facilitate systemic insulin signaling in fruit flies (Brankatschk et al., 2014). Thus, we decided to probe for the localization of the transcription factor dFOXO (Figure 4). dFOXO is the most downstream target of the insulin pathway and resides in the nucleus at low metabolic rates. We found that only microbe-infested larvae kept on CSP-F show a predominant nuclear dFOXO localization. In stark contrast, dFOXO in axenic CSP-F-feeding siblings is mainly cytoplasmic (Figure 4B, ANOVA test between CSP and ax CSP: *p* < 0.0001). In addition, we found that microbes do not change dFOXO activity in larval cultures thriving on SEP-F, SSP-F, or CEP-F (Figure 4B, ANOVA test between food type and its axenic counterpart: *p* > 0.05). Taken together, associated microbes block dFOXO activity in animals feeding on CSP-F. However, only axenic cultures kept on SSP-F match the developmental success shown by their microbe-infested counterparts (Figure 3).

**Figure 5:**
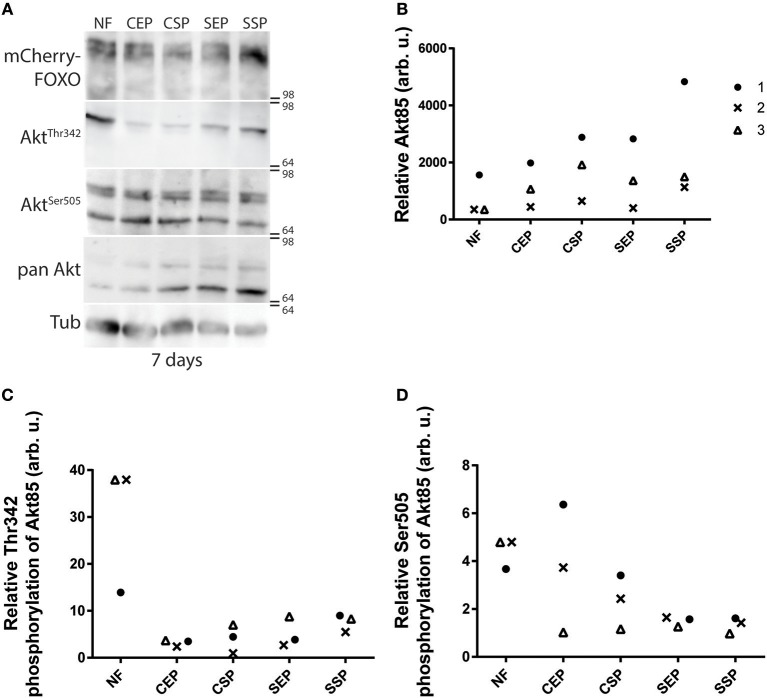
Individual dAkt isoforms facilitate dInR induced cellular insulin signaling. **(A)** Protein samples from flies kept on normal food (NF), and food from stationary (SP), or exponentially (EP) grown *S. cerevisiae* (S) or *C. oligophagum* (C). Shown is a photograph from a Western-Blot membrane of adult head-samples from flies taken 7 days after transfer from normal food (NF) to the respective yeast diet probed for mCherry (mCherry-FOXO), phosphorylated dAkt (Akt^Thr342^ or Akt^Ser505^), pan-dAkt protein (pan Akt), and Tubulin (Tub). **(B–D)** Quantification of three independent Western-Blot replica from head-samples (9 heads per sample) of adult flies taken 7 days after transfer from normal food (NF) to the respective yeast diet (SSP, SEP, CSP, or CEP). Shown are relative ratios of pan-dAkt^85^/Tubulin **(B)**, phosphorylated dAkt^85−Thr342^/pan-dAkt^85^ **(C)**, and dAkt^85−Ser505^/pan-dAkt^85^ **(D)**.

Many different dILPs regulate the larval development and thus, mimic the function of vertebrate insulin-like growth factors (IGFs; Brogiolo et al., 2001). Moreover, invertebrate dILPs probably represent an ancestral regulative network that channels IGFs and insulin-like signaling to one receptor (Barbieri et al., 2003; Grönke et al., 2010). Therefore, developmental dILPs blur metabolic aspects of the insulin circuit. To circumvent the problem, we decided to analyse dAkt activity in adult fly-head samples since dILPs expressed only in the larval and pupal stage are not present anymore (Zhang et al., 2009; Grönke et al., 2010). Phosphorylated active dAkt deactivates dFOXO (Calnan and Brunet, 2008). Therefore, we speculated with reference to the shown oviposition preference of mated females (Figure 2D) that only flies kept on SSP-F will show high dAkt phosphorylation levels. To test our hypothesis, we raised adults on normal food and then transferred the animals onto different yeast-based diets for 7 or 14 days at 20°C. We found that early into our experiment dFOXO levels do not change in flies kept on different diets (Figure 5A). Interestingly, compared with normal food-fed specimen, flies kept on our yeast-food types upregulate dAkt^85^ protein levels after 7 days (Figures 5A,B). Later, at the 14-day time point, only flies on SSP-F maintain higher or similar dAkt^85^ amounts with respect to normal food feeding animals (Supplementary Figures 4A,B). Interestingly, during the early upregulation of dAkt^85^, the relative phosphorylation levels at Akt^85−Ser505^ and Akt^85−Thr342^ drop with increasing protein amounts (Figures 5C,D). This trend is not continued; after 2 weeks, relative dAKT^85^ phosphorylation stabilizes close to levels shown by animals kept on normal food (Supplementary Figures 4C,D). Of note, dAkt^66^ is phosphorylated at its dAkt^66−Ser505^ position, but never detectable at dAkt^66−Thr342^ (Figure 5A, Supplementary Figure 4A). We conclude that in adult differentiated cells, dAkt^85^ is the predominant metabolically active dAkt isoform; and we propose that dietary lipids present in stationary *S. cerevisiae* elevate or stabilize dAkt^85^ amounts to facilitate cellular insulin signaling.

## Discussion

Environmental factors drive the spread and the dynamic behavior of insect populations as well as the quality and abundance of accessible food sources. Recent findings add another dimension to this simple equation, the identity and state of food-associated microbes (Nicholson et al., 2012). *Drosophila* are attracted by volatile yeast cues and prefer to feed on calorie-rich rotting plant material infested by yeast and other microbes (Becher et al., 2012). Such microbes share often the optimal temperature range of fruit flies (Watson, 1987; Petavy et al., 2001; Tsuji et al., 2017). Hence, available rotting fruits or other decomposing plant materials, in a given area, are infested by different yeast species (Chandler et al., 2012). We have isolated the yeast *Cystobasidium oligophagum* from wild type *OregonR* flies kept outside of the laboratory, a ubiquitous fungus known to degrade plant material. Is *C. oligophagum* a likely candidate associated with fruit flies? We show that *C. oligophagum* attracts flies and that *Drosophila* consume these yeasts. The fact that we have found this fungus in fly droppings indicates that some cells survive the intestinal passage (Supplementary Figure 5). However, to what proportion *C. oligophagum* is present on rotting fruits remains unclear and it remains to be shown if these type of yeast is found on wild flies (Chandler et al., 2012). In the laboratory, *Saccharomyces cerevisiae* is widely used to cultivate *Drosophila*. Recently, it was reported that lipid extracts from *S. cerevisiae* accelerate the generative cycle of flies by increasing the systemic insulin signaling rate (Brankatschk et al., 2014). However, *S. cerevisiae* are not predominant in regional natural habitats and it is likely that the interaction between fruit flies and their microbial surrounding is more complex (Hoang et al., 2015). In consequence, several questions arise in our quest to understand how microbes modulate the physiology and evolutionary conserved metabolic circuits of fruit flies. At first, we attempt to answer the question if all food-associated yeast provide factors that enhance the generative cycle of *Drosophila*.

At this point, it is not possible to estimate how natural variations of the genetic configuration in wild fly populations change the metabolic response of individuals. Therefore, we kept wild type flies (e.g., *OregonR* or *mCherry-FOXO*), harboring a defined genotype, on plant material supplemented with either *S. cerevisiae* or *C. oligophagum*. Interestingly, females feeding on *C. oligophagum* show low egg numbers compared to flies feeding on *S. cerevisiae*. Our finding allows for different explanations, either *C. oligophagum* is a poor diet or *C. oligophagum* products do not stimulate the metabolism of *Drosophila*. To exclude variables [including protein/sugar ratio, nutritional caloric load (Fanson et al., 2012), intestinal resorption (Diamond, 1991), microbial growth stage (Klose et al., 2012), or the immune response to microbes (Hoffmann, 2003)], we have created artificial food from both yeast. The resultant diets are enriched in protein and sugars to dwarf yields of respective fungal compounds. Of note, we found that the heat-treatment step in our food preparation protocols is changing the profile of hydrophobic yeast compounds detected by TLC. Reverse phase thin layer chromatography separates molecules based on their hydrophobicity and charge. Therefore, compounds like heat-sensitive vitamins containing fatty acid residues are well-detectable on TLC (Mohammad et al., 2012). To ensure the biological activity of our diets, we decided to repeat the egg-laying experiments performed earlier with living yeast. We observed that adult flies prefer *S. cerevisiae* food, especially produced from stationary cells. In addition, the same diet promoted development. Our results fit well with earlier experiments based on yeast food prepared from commercially available dry Baker's yeast from the grocery store (Carvalho et al., 2012; Brankatschk et al., 2014, 2018). Such dry yeast are composed from stationary cells grown in a fermenter. We speculate that the short reactivation time of dry yeast prior to our experiments (food preparation protocols, experiments with live yeast) is not sufficient to change their nutritional profile and therefore, *S. cerevisiae* harvested in their exponential growth phase nor any *C. oligophagum* culture accelerated *Drosophila* development.

Fly-associated microbes can modulate the nutritional input and, in some cases, promote directly or indirectly the insulin pathway. For instance, *Acetobacter pomorum* secrete metabolites capable to induce insulin signaling (Shin et al., 2011). In poor nutritional conditions, *Lactobacterium* replenish amino acid shortages and thus, restore the metabolic activity of the host (Storelli et al., 2011). To exclude the microbial variable in our experiments, we decided to test the development of microbe-free (axenic) larvae. Axenic animals tend to develop a metabolic syndrome, similar to insulin resistance, resulting in lower survival and slower developmental rates (Shin et al., 2011; Ridley et al., 2012). Therefore, we decided to run microbe-bearing and microbe-free cultures in parallel. Astonishingly, only axenic cultures thriving on stationary *S. cerevisiae* food matched the developmental parameters from siblings kept on the respective microbe-infested diet. We assume stationary yeast produce a heat-stable biological active compound capable to accelerate the development of *Drosophila*. We propose two possibilities; first, that lipid extracts from stationary yeast change the lipidome of the host leading to increased cellular insulin responsiveness. Although we did not detect any changes of endogenous complex lipids in response to the provided diets, our TLC-based analyses may not be sensitive enough. A better view could be provided by lipid mass spectroscopy (Carvalho et al., 2012) and the functional tests of biophysical membrane properties (Brankatschk et al., 2018). Second, it was shown that lipid extracts from stationary *S. cerevisiae* hyper-activate insulin-producing cells (Brankatschk et al., 2014). The consequent high circulating levels of insulin-like peptides stimulate the InR regulated cellular signal cascade (Brankatschk et al., 2014). We found that the transcription factor dFOXO changes its nuclear localization in response to the microbial load associated with the animals. It is widely accepted that the mTor-like signaling, one integral component of the *Drosophila* immune system, is modulating Akt and hence, changing FOXO activity (Pourrajab et al., 2015). Our cultivation protocol mildly stimulates the larval digestive system and food-associated microbes likely induce an immune response. Therefore, it is fair to speculate that developmental differences between axenic and non-axenic animals are not purely based on nutritional/metabolic factors (Slack et al., 2015).

Larval development includes the expression of many different dILPs that control the growth rate and morphological appearance of tissues irrespective of the metabolic input. To disentangle developmental from metabolic aspects, we resolved to study the phosphorylation of Akt, a central enzyme of the InR-controlled signal cascade, in adult flies. It is widely accepted that phosphorylated Akt is active, and that active Akt is responsible for the negative regulation of FOXO (Calnan and Brunet, 2008). Vertebrates express different Akt isoforms; for instance mice or humans express three different Akt proteins. Of note, the different isoforms are not equally expressed in all cell types (Gonzalez and McGraw, 2009a,b). Surprisingly, knock-out mice with only one functional copy of Akt1 are viable but show some physiological changes and are sensitive to dietary sugar loads (Dummler et al., 2006). However, most studies presume a parallel activity of all Akt isoforms and value the contribution of individual Akt isoforms in the insulin signal cascade based on their present levels (Gonzalez and McGraw, 2009a; Santi and Lee, 2010). Alike vertebrates, fruit flies express three different isoforms of the kinase Akt (Andjelković et al., 1995). As reported, we have detected two isoforms in our adult head-samples, dAkt^66^ and dAkt^85^. However, we found that dAkt^85^ is predominantly regulated in response to dietary cues and that dAkt^66^ is hardly phosphorylated at the dAkt^66−Thr342^ site. We conclude that individual dAkt isoforms in adult flies are differently addressed in response to dietary cues. In sum, we predict that lipid extracts from stationary *S. cerevisiae* increase circulating dILP levels and more insulin peptides bind to their receptor (Brankatschk et al., 2014). The insulin-bound dInR recruit additional PI3 kinases, which induce the production of Phosphoinositol-(3,4,5)-phosphate (PIP_3_) by conversion of membrane integrated Phosphoinositol-(4,5)-phosphate (PIP_2_) (Scanga et al., 2000). Therefore, the PIP2:PIP3 ratio is shifted and more cytoplasmic dAkt^85^ associates with PIP3 and is phosphorylated by local kinases. Further, phosphorylated dAkt^85^ regulates the downstream transcription factor dFOXO; this would implicate that dAkt^85^, similar to vertebrate Akt2 (Garofalo et al., 2003), is responsible for the insulin-mediated glucose disposal. In addition, since dAkt^66^ is never phosphorylated at its Thr342 position, we reinforce the idea that dAkt^66^, like vertebrate Akt3, fulfills more developmental roles (Andjelković et al., 1995; Santi and Lee, 2010) or is rather required to amplify signals from none-metabolic pathways (Weichhart and Saemann, 2008; Grootjans et al., 2016).

In sum, we add, to the complexity of microbe-host interactions, the variable of microbial growth stage; and we spotlight the differential phosphorylation and expression of dAkt isoforms as a versatile tool to facilitate the cellular insulin signal cascade. In addition, our work positions nutritional lipids as important metabolic modulators within a high-caloric environment; therefore, the quality of dietary lipids may represent a suitable target to define bioactive food.

## Materials and Methods

### Fly Stocks

If not stated otherwise, flies were kept at RT in a day/night cycle. *mCherry::foxo* from S. Eaton lab, *CantonS* from J.-C. Billeter's lab and *OregonR* were purchased from Bloomington stock center.

### Yeast

*Saccharomyces cerevisiae* (BY4741) was obtained from K. Ostermann and *Cystobasidium oligophagum* are wild isolates identified by M. Kaltenpoth.

### Food Recipes

Normal food (https://bdsc.indiana.edu/information/recipes/bloomfood.html) and plant food (Carvalho et al., 2012) were produced following published protocols, using dry-yeast purchased from the grocery discounter Kaufland. *Saccharomyces cerevisiae* (BY4741) and *C. oligophagum* foods were produced based on pelleted yeast obtained from SCP-medium (1.9 g/L yeast nitrogen base, 5 g/L ammonium sulfate, 20 g/L glucose, 20 g/L peptone) cultures grown at 20°C (v = 100 ml; wet yeast-pellet = 9.04 g, glucose = 6 g, soy peptone = 2 g, sucrose = 3 g, agar-agar = 1 g, and nipagin = 0.4 g). The Exponential Growth Phase (EP) is defined at the optical density OD_600_^EP^ = 2–3 for *S. cerevisiae* or a mass^EP^ of 0.02 g/ml for *C. oligophagum* and the Stationary Growth Phase (SP) at ${\text{OD}}_{600}^{\text{SP}}>$5 (*S. cerevisiae*) and a mass^SPof^ >0.04 g/ml (*C. oligophagum*). Of note, *C. oligophagum* cells tend to aggregate preventing a conventional optical density measurement.

### *Cystobasidium oligophagum* Isolation

*OregonR* were kept in an open cage on plant food without resistance factors for 2 weeks at 20°C, and subsequently transferred onto apple-juice plates. Deposited droppings were resolved in water and plated onto YPD-culture media plates. After 72 h at 20°C, microbial colonies were picked, cultivated in YPD media and samples send for 18S ribosomal sequencing.

### Behavior Assays

*CantonS* flies were raised on normal food, adults were transferred for 6 h on apple juice plates at 22°C, and subsequently placed on assay plates (20% apple juice, 1% agar, each plate with 3 males and 9 mated females) fitted with food baits opposing each other. Fly behavior was recorded for 3 h at 22°C and afterwards plates were kept for 24 h at 20°C. Subsequently, flies were removed, and deposed eggs counted.

### Larval Developmental Tracking

Eggs from *OregonR* collected from apple juice-agar plates were washed with tap water, bleached with Sodiumhypochloride (10%) and transferred onto apple juice plates. Later, hatched first-instar larvae were placed onto food and kept at 20°C.

### Axenic Animals

Eggs from *OregonR* collected from apple juice agar plates were washed with tap water, bleached with Sodiumhypochloride (10%) and transferred onto sterile apple juice plates (treated with hard UV light). Later, hatched first instar larvae were placed under sterile conditions onto microbe-free food using aseptic tools and kept at 20°C.

### Trehalose Measurements

Samples were autoclaved and processed like recommended by manufacturer (Trehalose Kit, Megazyme).

### Protein Estimation

Samples were autoclaved and processed like recommended by manufacturer (BCA Kit, Pierce).

### Lipid Extraction and TLC

Tissue samples were thawed on ice, homogenized in HBS using an IKA ULTRA-TURRAX disperser (level 5, 1 min), and lipid-extracted by the BUME method (Löfgren et al., 2012). Extracted lipids were stored in chloroform/methanol (2:1) solution at −80°C.

### Feeding Assay

Food intake measurements on dyed food was performed as described elsewhere (Deshpande et al., 2014). The food was stained with 1% w/v bromophenol blue solution. *mCherry::foxo* flies (one male and five mated females, 0 to 7-days old) were transferred on respective diets at 20°C. After 4 h, feeding was interrupted by freezing flies in liquid nitrogen. Only females were used to quantify blue stained fly guts.

### Biochemistry

#### Western Blot Analysis

*mCherry::foxo* flies were raised on normal food and 0 to 3-day-old adults were transferred for 14 days on respective diets at 20°C (male to female ratio 1:3; food renewal in regular intervals). Adults were snap-frozen with liquid nitrogen and fly heads removed. Fly heads were homogenized with a pestle in sample buffer (loading buffer, please see https://openwetware.org) and subsequently heat-inactivated for 10 min. Homogenates were centrifuged and supernatants used for TRIS-SDS-PAGE. Separated proteins were transferred by Tank-Blotting to nitrocellulose membrane. Membranes were blocked for 1 h with 5% BSA in 0.1% Triton X-100/PBS and then incubated with antibodies in blocking solution overnight. Polyclonal antibodies used to probe were Akt-pSer505 (Cell Signaling, 4054S), Akt-pThr308 (Invitrogen, 44-602G), Akt (Invitrogen, MAS14916), mCherry (Invitrogen, PAS-34974), and Tubulin (Cell Signaling, 2144S). After washing, membranes were incubated with HRP conjugated antibodies (Thermo Fischer, 31466) for 1 h and then bands detected with chemiluminescence.

#### Quantification

Samples were taken after 7 days (*n* = 3) and 14 days (*n* = 2) after specimen were transferred from normal food to respective yeast diets. Signals from Western blot membranes were photographed using an ImageQuant L4000 reader and photographs were analyzed using FIJI software (Schindelin et al., 2012). Each signal-band region of interest was selected with the “rectangle”-selection tool and the mean of fluorescence intensity was calculated. The intensity values have been normalized to the signals of the membrane background and of the tubulin signal from the same blots. Plotted are signal ratios between: dAKT^85^/Tubulin (Figure 5B, Supplementary Figure 4B), phospho-dAKT^85−Thr342^/dAKT^85^ (Figure 5C, Supplementary Figure 4C), and phospho-dAKT^85−Ser505^/dAKT^85^ (Figure 5D, Supplementary Figure 4D).

### Immunohistochemistry

#### Microscopy Analysis

*mCherry::foxo* and *CantonS* first instar larvae were transferred on respective food and kept at 20°C. After 5 days, larvae were fixed and dissected in 4% PFA, blocked for 1 h in blocking solution (5% native goat serum, 0.1% Triton X-100 in PBS, pH = 7.2) and incubated with anti-mCherry antibody (Invitrogen, PAS-34974) over night at 4°C. After washing, samples were incubated with goat-anti-rabbit-Alexa Fluor 555 (Thermo Fisher) and stained with DAPI for 2 h at room temperature. *mCherry*-FOXO signal in larval fat body was detected using confocal microscopy (Zeiss LSM700, 20x objective, respective Bandpass filters).

#### Quantification

The quantification of the mCherry signal has been performed on individual cells (cells) of larval fat bodies from several biological replicates (*n*). The densities of the fluorescence intensity (IntDen) were measured with Fiji Image J (Schindelin et al., 2012) (SEP: *n* = 8, cells = 41; SSP: *n* = 9, cells = 43; CEP: *n* = 9, cells = 45; CSP: *n* = 9, cells = 47; ax SEP: *n* = 9, cells = 48; ax SSP: *n* = 9, cells = 40; ax CEP: *n* = 3, cells = 15; ax CSP: *n* = 5, cells = 23). The IntDen values have been normalized to the IntDen signal values of *CantonS* fat bodies (*n* = 3–5, cells = 3–5). For each cell, the mCherry IntDen value of the whole cell (IntDen_cell_) and the nucleus (IntDen_nucleus_) have been measured by using FIJI and the “freehand”-selection tool; while the cytoplasm IntDen (IntDen_cyto_) signal has been calculated as following: IntDen_cyto_ = IntDen_cell_-IntDen_nucleus_ Then, the ratio IntDen_nucleus_/(IntDen_nucleus_ + IntDen_cyto_) have been calculated for each cell.

Yeast lipid droplets were stained with Bodipy 505/515 (Thermo Fisher) on ice for 2 h, and later life yeast were imaged using fluorescent-confocal microscopy (Zeiss LSM780, 63x oil objective, respective DIC, and Bandpass filters).

### Yeast Digestion Assay

*OregonR* third-instar larvae were transferred for 2 h into a tube containing Bodipy 505/515 stained *S. cerevisiae* or *C. oligophagum* (both harvested in stationary growth phase, grown at 20°C). Guts from feeding larvae were dissected in ice-cold PBS, and subsequently imaged using confocal microscopy (Zeiss LSM780, 10x objective, respective DIC, and Bandpass filters). Composite gut images were stitched together from overlapping single two-channel image tiles using the Fiji Plug in Preibisch et al. (2009).

### Statistics

Statistical analyses were performed with GraphPad Prism 7. Sample sizes are listed above or in figure legends. Difference between the mean ranks of blue stained food eating fly samples were calculated using Dunn's multiple comparisons test. Difference between the experimental groups for pupation speed and survival rate were calculated using Dunn's and Tukey's multiple comparisons test, respectively. Tukey's multiple comparisons test was also used to assess differences in nuclear mCherry-FOXO localization in fat body cells between groups.

## Data Availability Statement

All datasets generated for this study are included in the manuscript/Supplementary Files.

## Author Contributions

Experimental work by LT, EP, CM, NB, FH, and MG. Manuscript concept by LT, EP, MG, and MB.

### Conflict of Interest

The authors declare that the research was conducted in the absence of any commercial or financial relationships that could be construed as a potential conflict of interest.

## Supplementary Material

Supplementary Figure 1 Yeast growth state is important for lipid quality in *S. cerevisiae*. **(A,C)** Plotted are the growth curves of *S. cerevisiae* **(A)** or *C. oligophagum* in **(C)**. *R*^2^ indicates goodness of fit to the non-linear sigmoidal regression model. **(B,D)** Plotted are the protein and trehalose content of samples based on exponential (EP) and stationary (SP) *S. cerevisiae* (S) in **(B)** or *C. oligophagum* (C) in **(D)**. Lipid profiles **(E)** of polar and neutral lipids based on exponential (EP) and stationary (SP) *S. cerevisiae* (S) (SEP, SSP) or *C. oligophagum* (C) (CEP, CSP) were analyzed by 2-D Thin-layer chromatography.

Supplementary Figure 2 Heat-treatment changes the lipid profile of yeast. Shown is the lipid profile of yeast and yeast food lipid extract samples by separation with 1-D Thin-layer chromatography. Samples are based on sole stationary (SP) *S. cerevisiae* (S) or *C. oligophagum* (C) and respective foods (food), before and after autoclaving (aut.). Lipid markers include: CE, cholesterol esters; Cer-PE, ceramide phosphorylethanolamine; DAG, diacylglycerol; Erg, ergosterol; FA, fatty acid; MAG, monoacylglycerols; TAG, triacylglycerol.

Supplementary Figure 3 Fly endogenous lipid composition is not altered by nutritional lipids. **(A,B)** Shown is the separation of larval **(A)** and adult head **(B)** lipid extract samples by 1-D Thin-layer chromatography. Samples from animals kept on food based on exponential (EP) and stationary (SP) *S. cerevisiae* (S) or *C. oligophagum* (C). Samples were analyzed in triplicates (1–3). Lipid markers include: Card, cardiolipin; CE, cholesterol esters, Cer, ceramide; Ch-Ac, cholesterol-acetate; Chol, cholesterol; CPE, ceramide phosphorylethanolamine; DAG, diacylglycerol; Erg, ergosterol; FA, fatty acid; Gb3, globotriaosylceramide; GC, glucosylceramide; GM1-3, ganglioside; Lan, lanosterol; LC, lactosylceramide; PA, phosphatidic acid; PC, phosphatidylcholine; PE, phosphatidylethanolamine; PG, phosphatidylglycerol; PI, phosphatidylinositol; POPG, 2-Oleoyl-1-palmitoyl- sn-glycero-3-phospho-rac-(1-glycerol); PS, phosphatidylserine; Sit, sitosterol; SM, sphingomyelin; TAG, triacylglycerol.

Supplementary Figure 4 Individual dAkt isoforms facilitate dInR induced cellular insulin signaling. **(A)** Protein samples from flies kept on normal food (nf), and food from stationary (sp) or exponentially (ep) grown *S. cerevisiae* (s) or *C. oligophagum* (c). Shown is a photograph from a Western-Blot membrane of adult head-samples from flies taken 14 days after transfer from normal food (NF) to the respective yeast diet probed for mCherry-dFOXO, p-dAkt (Akt^Thr342^ or Akt^Ser505^), pan-dAkt protein (Akt), and Tubulin (Tub). **(B–D)** Quantification of two independent Western-Blot replica from head-samples (*n* = 9 heads each sample) of adult flies taken 14 days (14d_n) after transfer from normal food (NF) to the respective yeast diet (SSP, SEP, CSP, or CEP). Shown are relative ratios of pan-dAkt^85^/Tubulin **(B)**, phosphorylated dAkt^85−Thr342^/pan-dAkt^85^ **(C)**, and dAkt^85−Ser505^/pan-dAkt^85^ **(D)** with respect to samples from specimen kept on normal food.

Supplementary Figure 5 Some *Cystobasidium* cells might survive the larval intestinal passage. **(A,B)** Third instar larvae were fed with stained (Bodipy) *S. cerevisiae* **(A)** or *C. oligophagum* **(B)**. Guts from feeding larvae were dissected and imaged. Shown is a stitched whole gut image and two distinct magnified sections, part of the anterior midgut (1) and hindgut (2). Black scale bars = 500 μm, white scale bars = 50 μm.

Supplementary Movies Exemplary movies show the behavior of adult flies on plates. Food samples are based on exponential (EP) and stationary (SP) *S. cerevisiae* (SEP, SSP) or *C. oligophagum* (CEP, CSP).

Movie 1 Testing plant food vs. plant food.

Movie 2 Testing *S. cerevisiae* vs. plant food.

Movie 3 Testing *C. oligophagum* vs. plant food.

Movie 4 Testing SEP vs. plant food.

Movie 5 Testing SSP vs. plant food.

Movie 6 Testing CEP vs. plant food.

Movie 7 Testing CSP vs. plant food.
