# Supplementary figures and images for: Selective Phosphorylation of Akt/Protein-Kinase B Isoforms in Response to Dietary Cues
Source: Front Cell Dev Biol. 2019 Oct 10;7:206. doi: 10.3389/fcell.2019.00206 (PMC6796796; doi:10.3389/fcell.2019.00206)

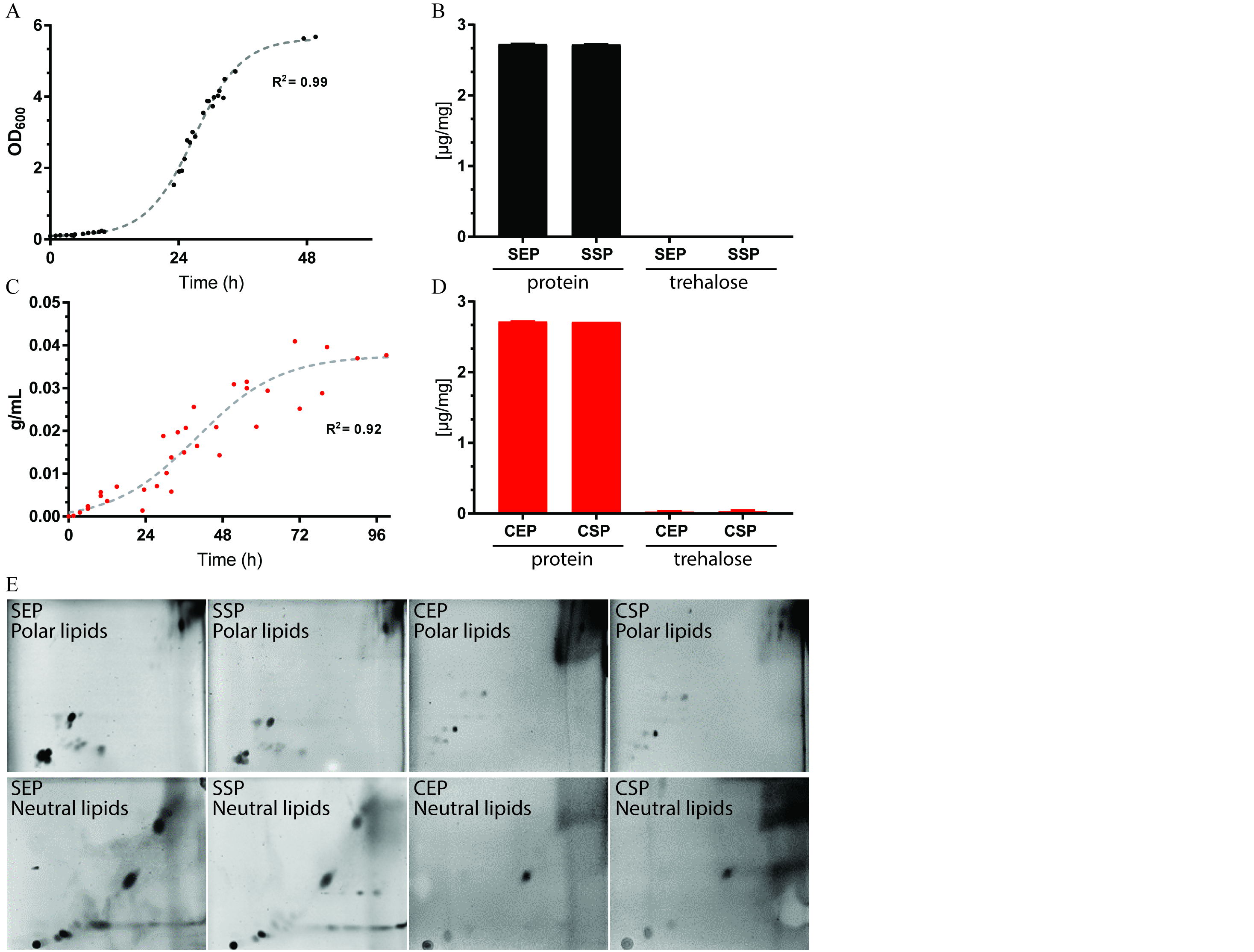

Supplement: Supplementary Figure 1 — Yeast growth state is important for lipid quality in S. cerevisiae. (A,C) Plotted are the growth curves of S. cerevisiae (A) or C. oligophagum in (C). R2 indicates goodness of fit to the non-linear sigmoidal regression model. (B,D) Plotted are the protein and trehalose content of samples based on exponential (EP) and stationary (SP) S. cerevisiae (S) in (B) or C. oligophagum (C) in (D). Lipid profiles (E) of polar and neutral lipids based on exponential (EP) and stationary (SP) S. cerevisiae (S) (SEP, SSP) or C. oligophagum (C) (CEP, CSP) were analyzed by 2-D Thin-layer chromatography. [file Image_1.TIF]

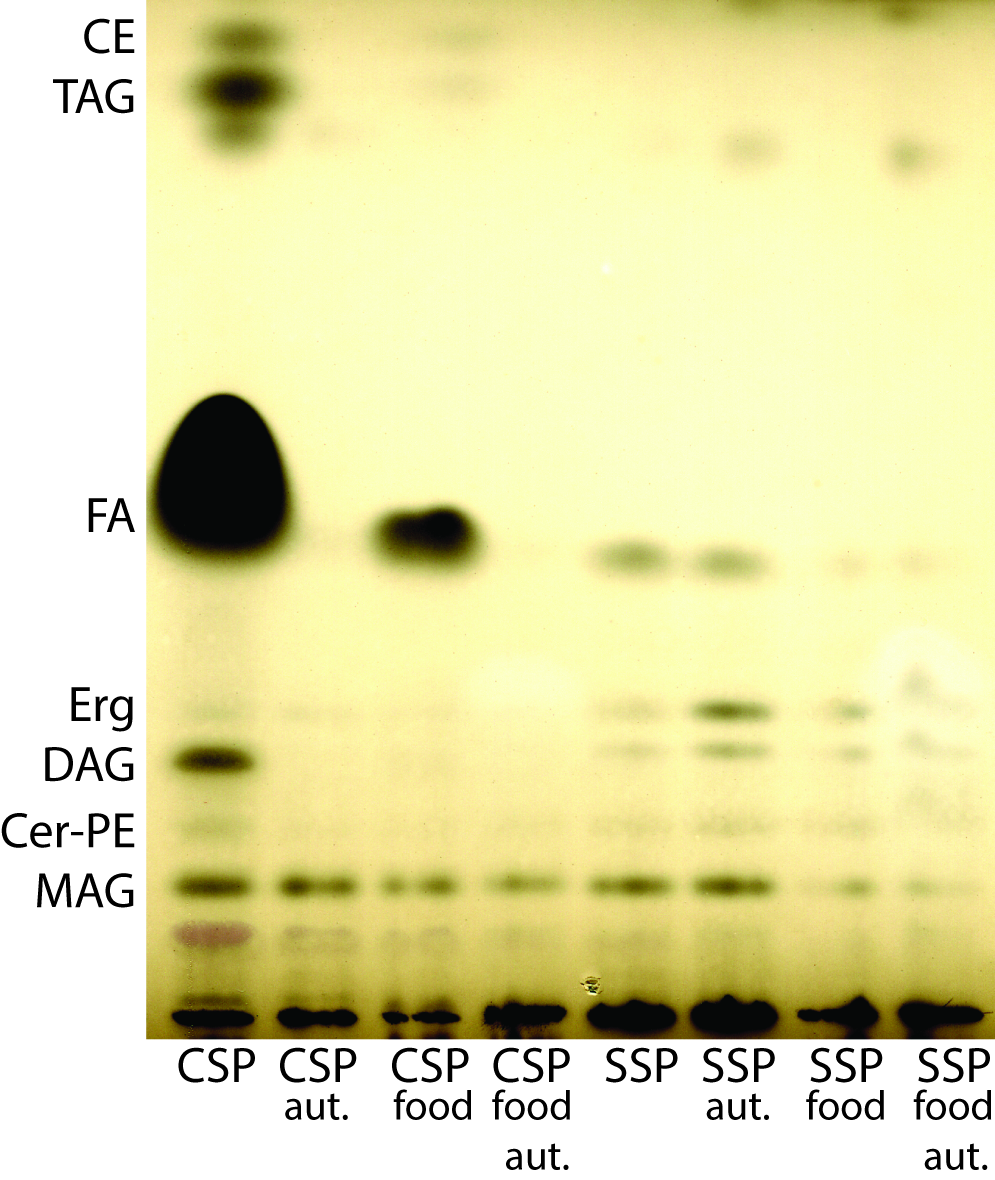

Supplement: Supplementary Figure 2 — Heat-treatment changes the lipid profile of yeast. Shown is the lipid profile of yeast and yeast food lipid extract samples by separation with 1-D Thin-layer chromatography. Samples are based on sole stationary (SP) S. cerevisiae (S) or C. oligophagum (C) and respective foods (food), before and after autoclaving (aut.). Lipid markers include: CE, cholesterol esters; Cer-PE, ceramide phosphorylethanolamine; DAG, diacylglycerol; Erg, ergosterol; FA, fatty acid; MAG, monoacylglycerols; TAG, triacylglycerol. [file Image_2.TIF]

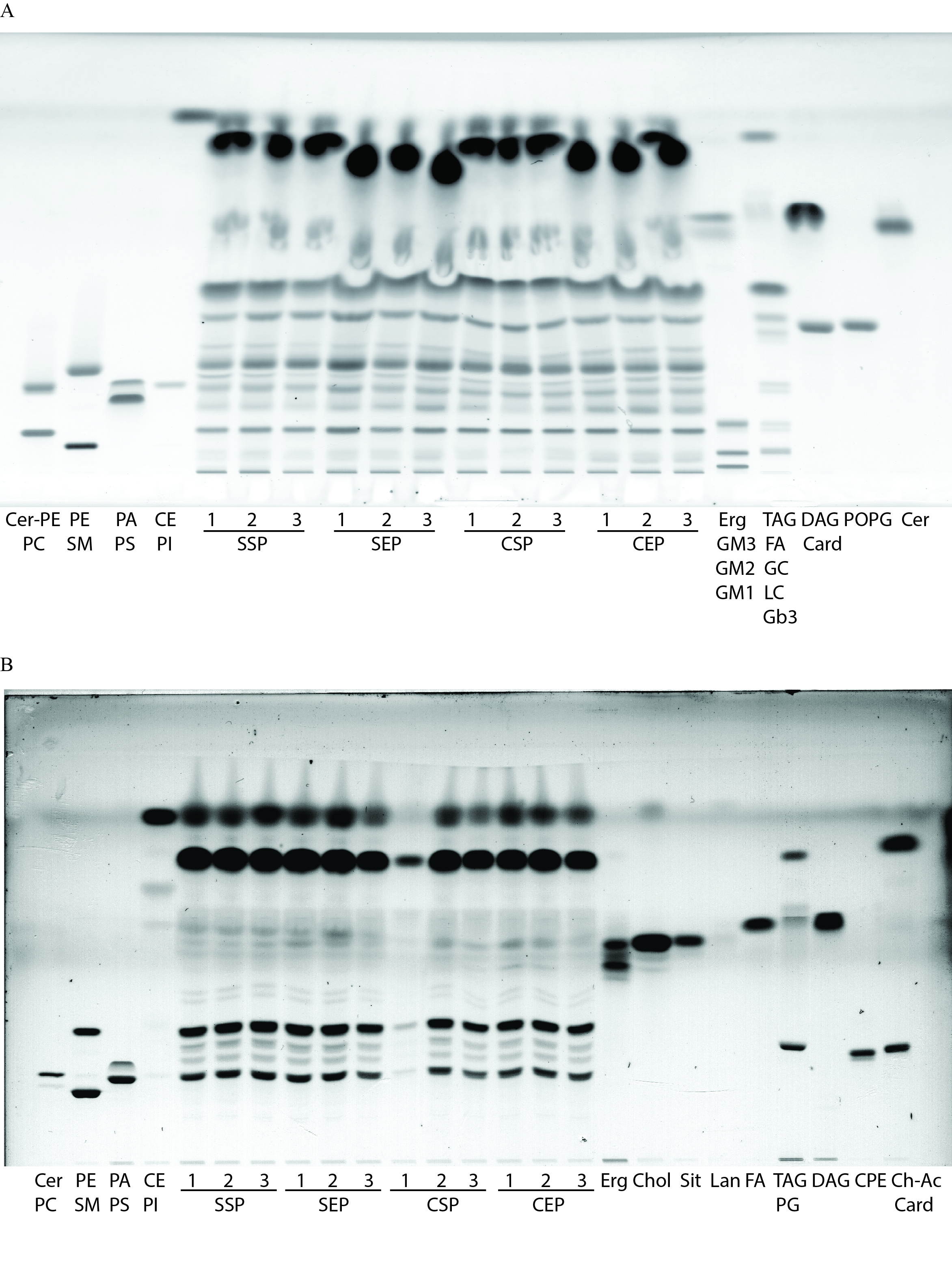

Supplement: Supplementary Figure 3 — Fly endogenous lipid composition is not altered by nutritional lipids. (A,B) Shown is the separation of larval (A) and adult head (B) lipid extract samples by 1-D Thin-layer chromatography. Samples from animals kept on food based on exponential (EP) and stationary (SP) S. cerevisiae (S) or C. oligophagum (C). Samples were analyzed in triplicates (1–3). Lipid markers include: Card, cardiolipin; CE, cholesterol esters, Cer, ceramide; Ch-Ac, cholesterol-acetate; Chol, cholesterol; CPE, ceramide phosphorylethanolamine; DAG, diacylglycerol; Erg, ergosterol; FA, fatty acid; Gb3, globotriaosylceramide; GC, glucosylceramide; GM1-3, ganglioside; Lan, lanosterol; LC, lactosylceramide; PA, phosphatidic acid; PC, phosphatidylcholine; PE, phosphatidylethanolamine; PG, phosphatidylglycerol; PI, phosphatidylinositol; POPG, 2-Oleoyl-1-palmitoyl- sn-glycero-3-phospho-rac-(1-glycerol); PS, phosphatidylserine; Sit, sitosterol; SM, sphingomyelin; TAG, triacylglycerol. [file Image_3.JPEG]

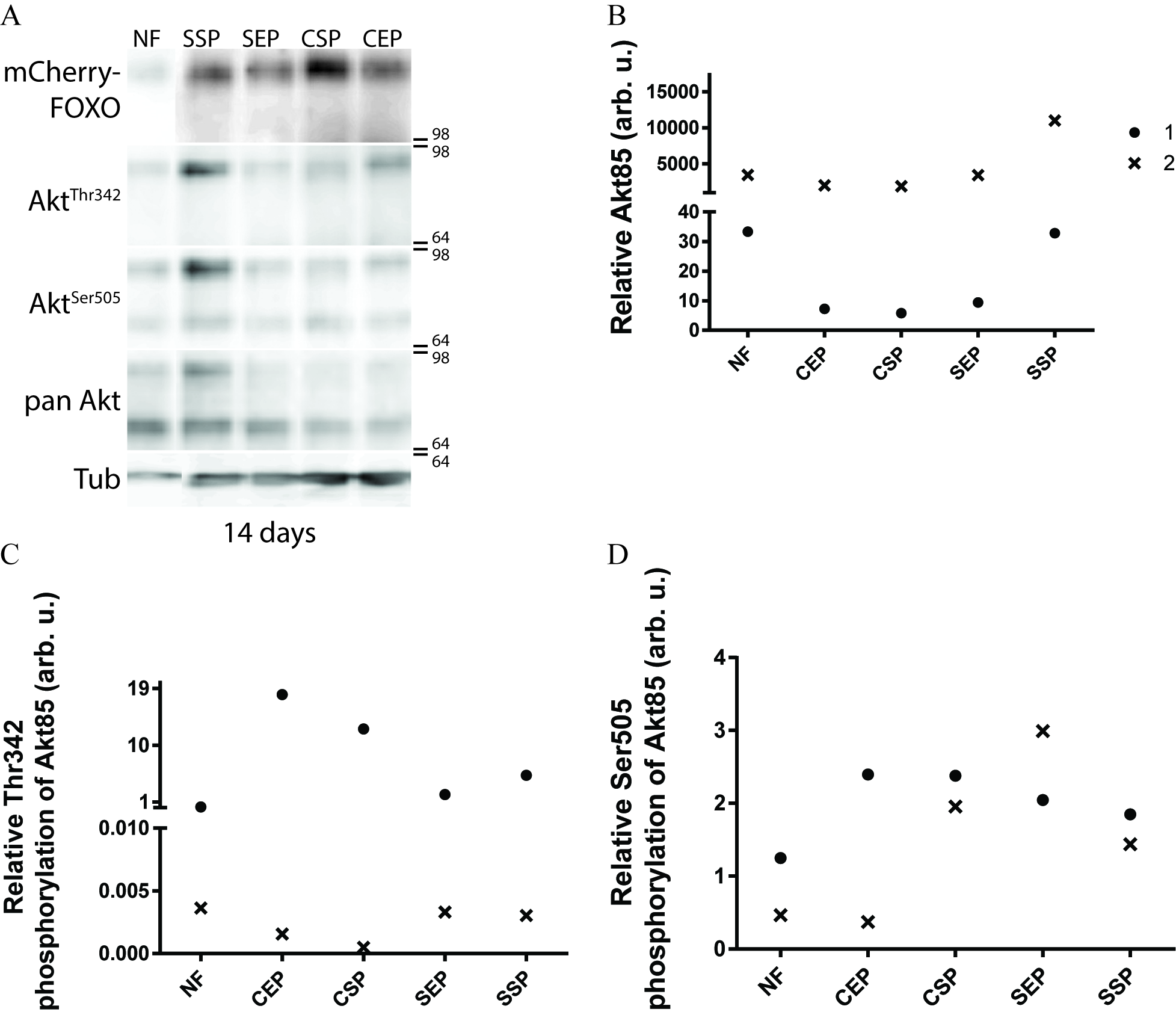

Supplement: Supplementary Figure 4 — Individual dAkt isoforms facilitate dInR induced cellular insulin signaling. (A) Protein samples from flies kept on normal food (nf), and food from stationary (sp) or exponentially (ep) grown S. cerevisiae (s) or C. oligophagum (c). Shown is a photograph from a Western-Blot membrane of adult head-samples from flies taken 14 days after transfer from normal food (NF) to the respective yeast diet probed for mCherry-dFOXO, p-dAkt (AktThr342 or AktSer505), pan-dAkt protein (Akt), and Tubulin (Tub). (B–D) Quantification of two independent Western-Blot replica from head-samples (n = 9 heads each sample) of adult flies taken 14 days (14d_n) after transfer from normal food (NF) to the respective yeast diet (SSP, SEP, CSP, or CEP). Shown are relative ratios of pan-dAkt85/Tubulin (B), phosphorylated dAkt85−Thr342/pan-dAkt85 (C), and dAkt85−Ser505/pan-dAkt85 (D) with respect to samples from specimen kept on normal food. [file Image_4.TIF]

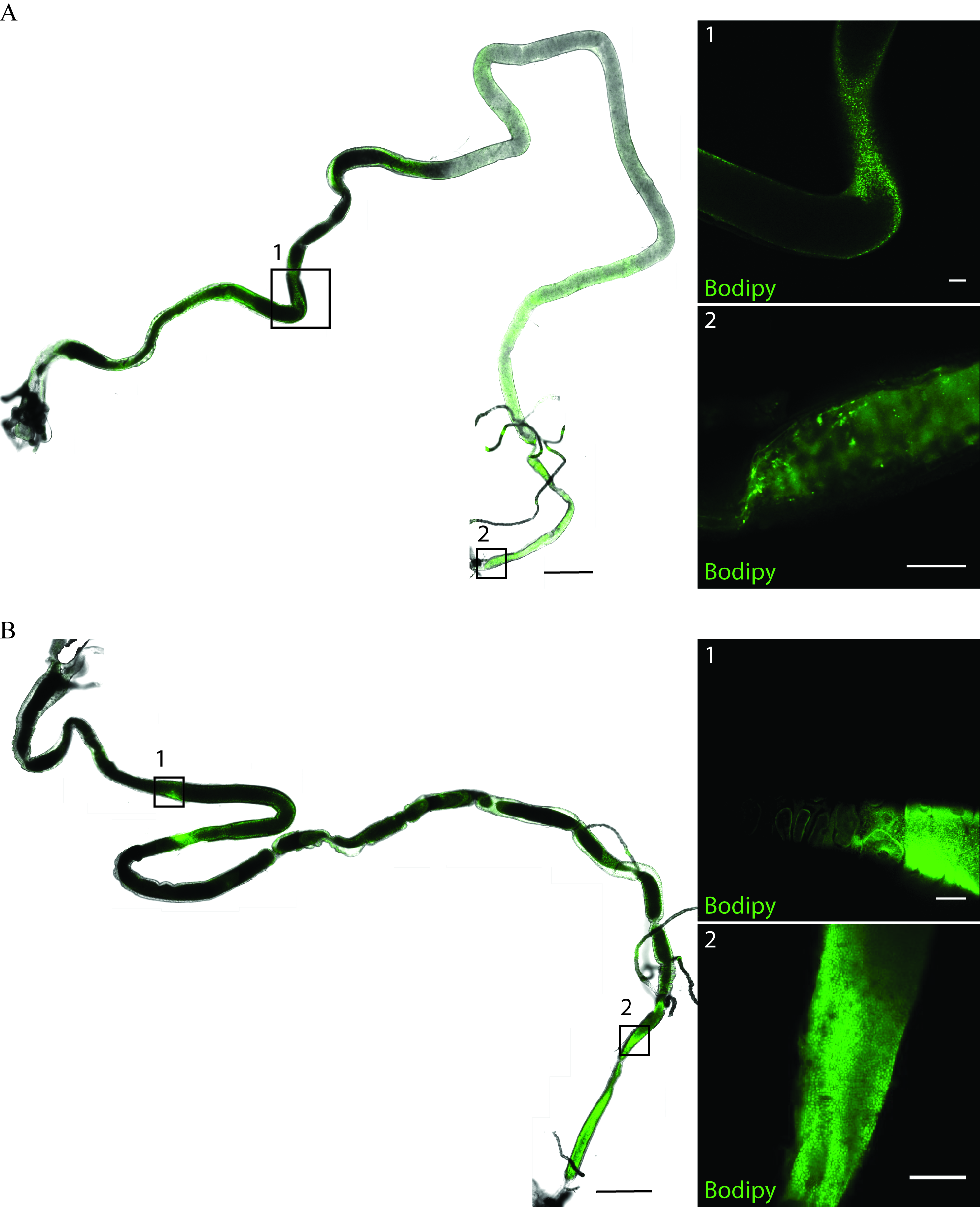

Supplement: Supplementary Figure 5 — Some Cystobasidium cells might survive the larval intestinal passage. (A,B) Third instar larvae were fed with stained (Bodipy) S. cerevisiae (A) or C. oligophagum (B). Guts from feeding larvae were dissected and imaged. Shown is a stitched whole gut image and two distinct magnified sections, part of the anterior midgut (1) and hindgut (2). Black scale bars = 500 μm, white scale bars = 50 μm. [file Image_5.TIF]
